# Phylogenetic relationships and genetic diversity of the Korean endemic *Phedimus latiovalifolius* (Crassulaceae) and its close relatives

**DOI:** 10.1038/s41598-024-63272-9

**Published:** 2024-07-15

**Authors:** Myong-Suk Cho, Yongsung Kim, Seon-Hee Kim, Ji-Hyeon Jeon, JiYoung Yang, Seung-Chul Kim

**Affiliations:** 1 Department of Biological Sciences, Sungkyunkwan University, Suwon, 16419 Republic of Korea; 2 Honam National Institute of Biological Resources, Mokpo, 58762 Korea; 3 Department of Botany, Graduate School of Science, Kyoto University, Sakyo-Ku, Kyoto, 606-8502 Japan; 4 Institute for Dok-Do and Ulleung-Do Island, Kyungpook National University, Daegu, 41566 Republic of Korea

**Keywords:** Genetic diversity, Crassulaceae, Single nucleotide polymorphisms, Glacial refugium, Baekdudaegan, Korean, Evolution, Plant sciences

## Abstract

*Phedimus latiovalifolius* (Y.N.Lee) D.C.Son & H.J.Kim is exclusively distributed in the high mountains in the Korean Peninsula, mainly along the Baekdudaegan mountain range. Despite its morphological and distributional distinction from other *Phedimus* Raf. species, its taxonomic identity and phylogenetic relationship with congeneric species remain unclear. This study employs genotyping-by-sequencing-derived genome-wide single nucleotide polymorphisms to establish the monophyly of *P. latiovalifolius* and its relationship with closely related species. Genetic diversity and population differentiation of *P. latiovalifolius* are also assessed to provide baseline genetic information for future conservation and management strategies. Our phylogenetic analyses robustly demonstrate the monophyletic nature of *P. latiovalifolius*, with *P. aizoon* (L.) ‘t Hart identified as its closest sister lineage. There is no genetic evidence supporting a hybrid origin of *P. latiovalifolius* from *P. aizoon* involving either *P. ellacombeanus* (Praeger) ‘t Hart or *P. kamtschaticus* (Fisch.) ‘t Hart. Population genetic analyses reveal two major groups within *P. latiovalifolius*. A higher genetic variation is observed in *P. ellacombeanus* than in the congeneric species. Notably, most of the genetic variation exists within *P. latiovalifolius* populations. Given its distribution and the potential role of Baekdudaegan as an East Asian Pleistocene refugia, *P. latiovalifolius* could be considered rare and endemic, persisting in the refugium across glacial/interglacial cycles.

## Introduction

The genus *Phedimus* Raf.^1^ encompasses a group of succulent stonecrop plants within the family Crassulaceae, comprising approximately 20 species globally. Traditionally, *Phedimus* was classified under the broad genus *Sedum* L., however, this genus segregated from *Sedum*, because the species within *Phedimus* are distinguished by flattened leaves featuring serrate or crenate margins and longitudinally costate or sub-smooth testa. In contrast, *Sedum* species exhibit terete or semiterete leaves with entire margins and reticulate or papillate-reticulate testa^2–4^. Molecular phylogenetic studies consistently support the monophyly of *Phedimus* and its separation from *Sedum* sensu stricto^3–7^. Within the genus *Phedimus*, two subgeneric groups are recognized: *Phedimus* and *Aizoon* (L.K.A. Koch ex Schönland) Ohba & Turland^5,6^. Subgenus *Phedimus* comprises approximately five species with purple or white petals occurring in the Eurasian regions from the Aegean to South Persia and North Caucasus. These species are mainly diploid, except for *P. spurius* (M.Bieb.) ‘t Hart, exhibiting a simple descending dysploidy series with base chromosome numbers (x) ranging from x = 7, 6, to 5^2,8^. In contrast, subgenus *Aizoon* includes between 12 and 15 species with yellow petals ranging from East Europe in the South Urals to the Far East. *Aizoon* species have a base chromosome number of x = 8, with extensive polyploidy and aneuploidy reported^9–12^.

The subgenus *Aizoon* exhibits distinct morphological and karyological characteristics, yet the differentiation of its component species poses challenges due to the uniformity of floral traits and significant variability in vegetative features complicated further by extensive polyploidy and aneuploidy. This has led to intricate issues in defining species and understanding interspecific relationships^9–13^. In the context of Korea, species recognition within subgenus *Aizoon* has been inconsistent among authors, ranging from one^14^ to six^15^ species. Presently, eight species are recognized in Korea, constituting nearly half of the 15 species in subgenus *Aizoon*. This includes the recent recognition of *P. latiovalifolius*^16^, previously misreported as *P. latiovalifolium*^17^. The identified species are *P. aizoon*, *P. ellacombeanus*, *P. kamtschaticus*, *P. middendorffianus* (Maxim.) 't Hart, *P. takesimensis* (Nakai) 't Hart, *P. zokuriensis* (Nakai) 't Hart, and *P. daeamensis* T.Y.Choi & D.C.Son. Notably, five of these species are narrow endemics with limited geographical distributions: the evergreen creeping *P. takesimensis* on Ulleung Island, *P. zokuriensis* and *P. latiovalifolius* in central Korea, *P. daeamensis* in Mt. Daeam (Gangwon-do Province), and *P. ellacombeanus* in southern Korea. In contrast, P. *aizoon* and *P. kamtschaticus* are widely distributed throughout the Korean Peninsula, as well as in Russia, China, Mongolia, and Japan. *P. middendorffianus*, found in northern Korea, exhibits a broader distribution throughout Russia, China, and Japan.

*Phedimus latiovalifolius* is exclusively found in an elevation of approximately 1000 m above sea level (a.s.l.) within the narrow confines of the east-central Korean Peninsula. This region is part of the main mountain range and watershed crest line known as the Baekdudaegan (hereafter referred to as the 'BDDG'). The BDDG, which contains 14 subsidiary mountain ranges, runs through almost the entire peninsula for over 1400 km (701 km within South Korea), stretching from Mt. Baekdu (alt. 2744 m) to the north to Mt. Jiri (alt. 1915 m) to the south^18^. Although it occupies only approximately 2.6% of the total land and 4% of the total forest area of the South Korean Peninsula, the BDDG is a well-known biodiversity hotspot with high species richness and endemism, harboring approximately one-third (ca. 1326 species in the South Korean part of this mountain range) of the total flora of the Korean Peninsula. Of nearly 1300 species, 108 native to the BDDG are endemic to the Korean Peninsula, and 56 rare species are listed around the BDDG^19^.

**Figure 1:**
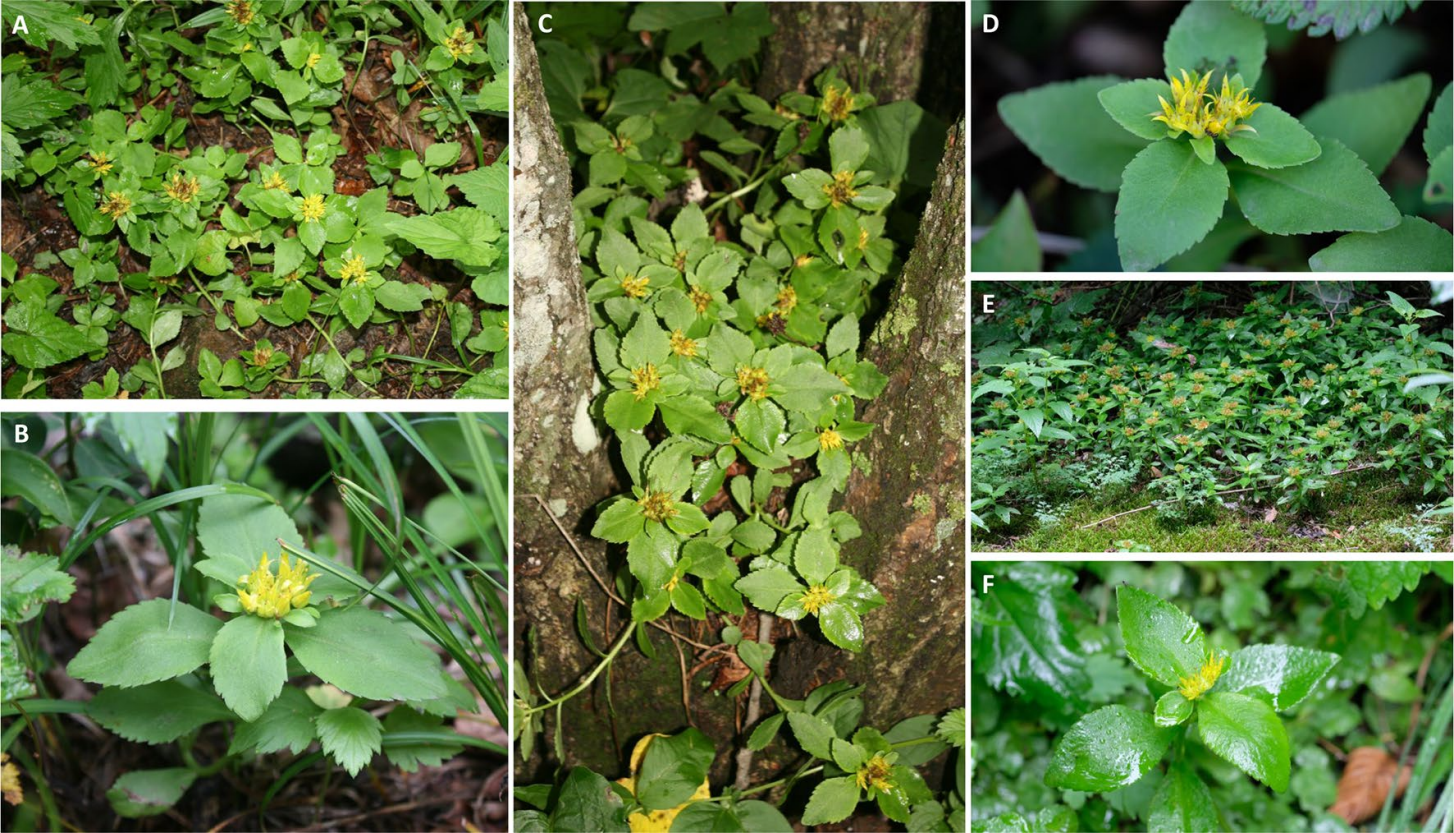
Photographs of the *Phedimus latiovalifolius* found in the type locality, Geumdaebong Peak (A-C; 1345 m a.s.l.), and two other localities, Mt. Dosol (D; 1029 m a.s.l.) and Mt. Seorak (E and F; 1010 m a.s.l.). Photo credit: Seung-Chul Kim.

**Figure 2:**
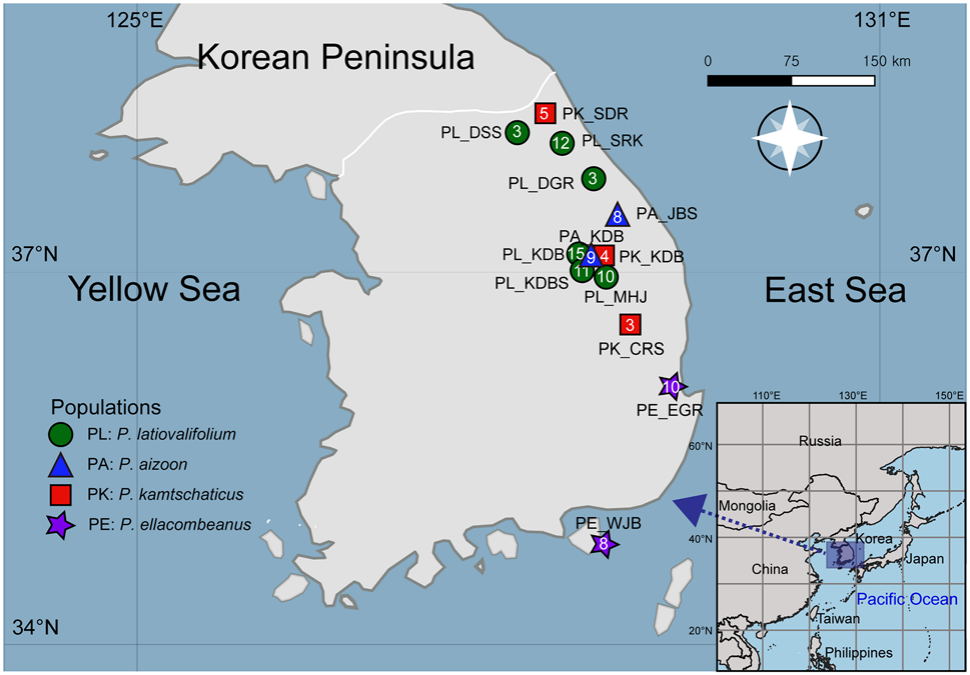
Geographic distributions of the *Phedimus latiovalifolius* populations and sympatric *Phedimus* species. Populations are coded by different icons and colors representing the sampling localities, species, and sizes (see Table 1 for population codes).

Initially described as *Sedum latiovalifolium* Y. Lee, *P. latiovalifolius* was identified on Geumdaebong Peak on Mt. Taebaek, Korea, at an altitude of around 1300 m a.s.l. The distinctiveness of *P. latiovalifolius* was recognized by Lee (1992)^16^, who highlighted its unique features such as the broadly ovate leaves forming a rosette on the terminal stem (Fig. 1) and its restricted distribution to Geumdaebong (Figs. 1A–C and 2), although he suggested its alliance to *Sedum ellacombeanum* (≡ current *P. ellacombeanus*). Lee^20^ later proposed a potential hybrid origin hypothesis involving *Sedum aizoon* (≡ *P. aizoon*) and *S. ellacombeanum* (≡ *P. ellacombeanus*) or *S. kamtschaticum* (≡ *P. kamtschaticus*), as *P. aizoon* and *P. kamtschaticus* coexist with *P. latiovalifolius* on Geumdaebong Peak. While *P. latiovalifolius* is limited to elevations above 1000 a.s.l. within a narrow range in Korea, its putative parental species, *P. aizoon*, and *P. kamtschaticus*, exhibit wide altitudinal and geographical ranges in East Asia^21,22^.

Once considered synonymous with *P. ellacombeanus* (Praeger) 't Hart^8^, *P. latiovalifolius* was only recently recognized as a distinct Korean endemic species. Morphometric analysis of *Phedimus* species in Korea did not initially identify *P. latiovalifolius* as a distinct species, associating it with *P. kamtschaticus* in the UPGMA phenogram^23^. *P. kamtschaticus* displayed varied morphological characteristics with a wide range of geographic and ecological distribution clustered with few other *Phedimus* species (*P. zokuriensis*, *P. ellacombeanus*, and *P. takesimensis*). *P. latiovalifolius* revealed the morphological closeness to the southern population of *P. kamtschaticus*, while it was distantly placed from *P. aizoon*. Yoo and Park^24^ refuted the hybrid origin of *P. latiovalifolius* between *P. aizoon* and *P. kamtschaticus* based on morphology (18 characters) and isozyme data (ten isozyme loci). *P. latiovalifolius* neither clustered with its putative parental species, *P. aizoon* or *P. kamtschaticus* in phenetic analysis, nor shared the high-frequency marker alleles with them in isozyme data. Recently, Moon and Jang^25^ reported that *P. latiovalifolius, P. takesimensis*, and *P. middendorffianus* should be recognized as distinct taxa based on their morphological characteristics. Molecular phylogenetic studies on interspecific relationships within the subgenus *Aizoon* species in Korea have encountered challenges in establishing robust phylogenetic relationships^26,27^. For instance, Seo et al. (2020)^26^ conducted a phylogenetic analysis of five Korean taxa (*P. takesimensis*, *P. aizoon*, *P. kamtschaticus*, *P. ellacombeanus*, and *P. latiovalifolius*) to assess the anagenetic speciation of *P. takesimensis* on Ulleung Island. However, the overall lack of resolution and support, along with insufficient sampling, prevented the determination of the closest sister species to *P. latiovalifolius*. Recently, Kim et al. (2023)^27^ explored overall species relationships within the subgenus *Aizoon* using complete plastomes and nrDNA ITS sequences. Despite these efforts, neither the monophyly of *P. latiovalifolius* nor the phylogenetic relationships within *Aizoon* were adequately addressed, attributed to insufficient sampling and/or limited tree resolution and node support.

The main goal of this study was to investigate the monophyly of *P. latiovalifolius* and determine its origin by inferring its phylogenetic relationships with closely related sympatric congeneric species. This study included the populations of the putative parental species, *P. aizoon*, *P. kamtschaticus*, and *P. ellacombeanus* as well as the populations of *P. latiovalifolius* to test its hybrid origin hypothesis suggested by Lee^20^ (See Fig. 2 and Table 1 for sampling details). In addition, we estimated the genetic diversity and population differentiation of *P. latiovalifolius* and other *Phedimus* species, utilizing 6642 genotyping-by-sequencing (GBS)-derived single nucleotide polymorphisms (SNPs) at the genome-wide level. GBS involves restriction enzymes for genome complexity reduction and next-generation sequencing (NGS) for SNP discovery and genotyping^28^. GBS method is suitable for population, taxonomic, and phylogenetic studies^29,30^, offering a cost-effective, high-throughput sequencing method for various species, including apple, barley, *Brassica*, maize, rice, wheat, and chickpea^31–37^. The findings from this study provide essential genetic information for establishing conservation and management strategies for highly vulnerable mountain species affected by climate change in Korea.

**Table 1:** Populations and collection sites of *P*. *latiovalifolius* and sympatric *Phedimus* species used in this study.

Species	Number	Sample ID	Population code/Voucher info	Locality	gps
*P. aizoon*	8	PA618201,2,3,5,6,7,8,9	PA_JBS/*SKK_PA200618201*	Jangbyeong Mountain, Samcheok-si, Gangwon-do, Korea	37°20′41.3″N 128°55′21.4″E, alt. 703 m
*P. aizoon*	9	PA626100,2,3,4,5,6, 7,8,9	PA_KDB/ *SKK_PA200626105*	Geumdaebong Peak, Taebaek Mountain, Jeongseon-gun, Gangwon-do	37°12′45.3″N 128°54′47.0″E, alt. 1345 m
*P. ellacombeanus*	8	PE609001,2,5,6,7,8, PE609500,1	PE_WJB/ *SKK_PE200609001*	Wujebong Peak, Geoje-si, Gyeongsangnam-do	34°43′48.7″N 128°40′30.9″E, alt. 92 m
*P. ellacombeanus*	10	PE615001,2,3,4,5,6, 7,8,9,10	PE_EGR/ *SKK_PE200615001*	Egari, Pohang-si, Gyeongsangbuk-do	36°11′06.8″N 129°22′55.6″E, alt. 21 m
*P. kamtschaticus*	5	PK528500,1,2,4,5	PK_SDR/ *SKK_PK200528500*	Sottongnyeong, Goseong-gun, Gangwon-do	38°19′55.5″N 128°22′42.3″E, alt. 98 m
*P. kamtschaticus*	3	PK623101,2,3	PK_CRS/ *SKK_PK200623101*	Cheongryang Mountain, Bonghwa-gun, Gyeongsangbuk-do	36°48′43.7″N 128°53′02.0″E, alt. 200 m
*P. kamtschaticus*	4	PK626500,2,3,4	PK_KDB/ *SKK_PK200626500*	Geumdaebong Peak, Taebaek Mountain, Jeongseon-gun, Gangwon-do	37°12′45.3″N 128°54′47.0″E, alt. 1345 m
*P. latiovalifolius*	3	PL620503,4,5	PL_DGR/ *SKK_PL200620503*	Daegwallyeong Pass, Pyeongchang-gun, Gangwon-do	37°41′47.9″N 128°45′21.6″E, alt. 952 m
*P. latiovalifolius*	15	PL626201,3,4,5,6, 8,9,12,15, PL528026, 7,8,9,30,31	PL_KDB/ *SKK_PL200626201*	Geumdaebong Peak, Taebaek Mountain, Jeongseon-gun, Gangwon-do	37°12′45.3″N 128°54′47.0″E, alt. 1345 m
*P. latiovalifolius*	11	PL528015,6,7,9,20, 21,22,23,24,25, PL528508	PL_KDBS/ *SKK_PL200528015*	Geumdaebong Peak south, Taebaek Mountain, Jeongseon-gun, Gangwon-do	37°12′20.7″N 128°54′55.3″E, alt. 1311 m
*P. latiovalifolius*	10	PL626700,1,2,5,6, 9,11,12,13,14	PL_MHJ/ *SKK_PL200626700*	Manhangjae, Hambaek Mountain, Jeongseon-gun, Gangwon-do	37°08′54.1″N 128°54′12.2″E, alt. 1297 m
*P. latiovalifolius*	3	PL701805,8,9	PL_DSS/ *SKK_PL200701805*	Dosol Mountain, Yanggu-gun, Gangwon-do	38°14′32.2″N 128°05′33.6″E, alt. 1029 m
*P. latiovalifolius*	12	PL709001,2,3,4,5,7, 8,11,13,14,15,16	PL_SRK/ *SKK_PL200709015*	Seorak Mountain, Yangyang-gun, Gangwon-do	38°06′12.8″N 128°27′25.9″E, alt. 1010 m
Total	101 Individuals		13 Populations		

## Results

### Phylogenetic trees derived from SNPs of GBS analysis

**Figure 3:**
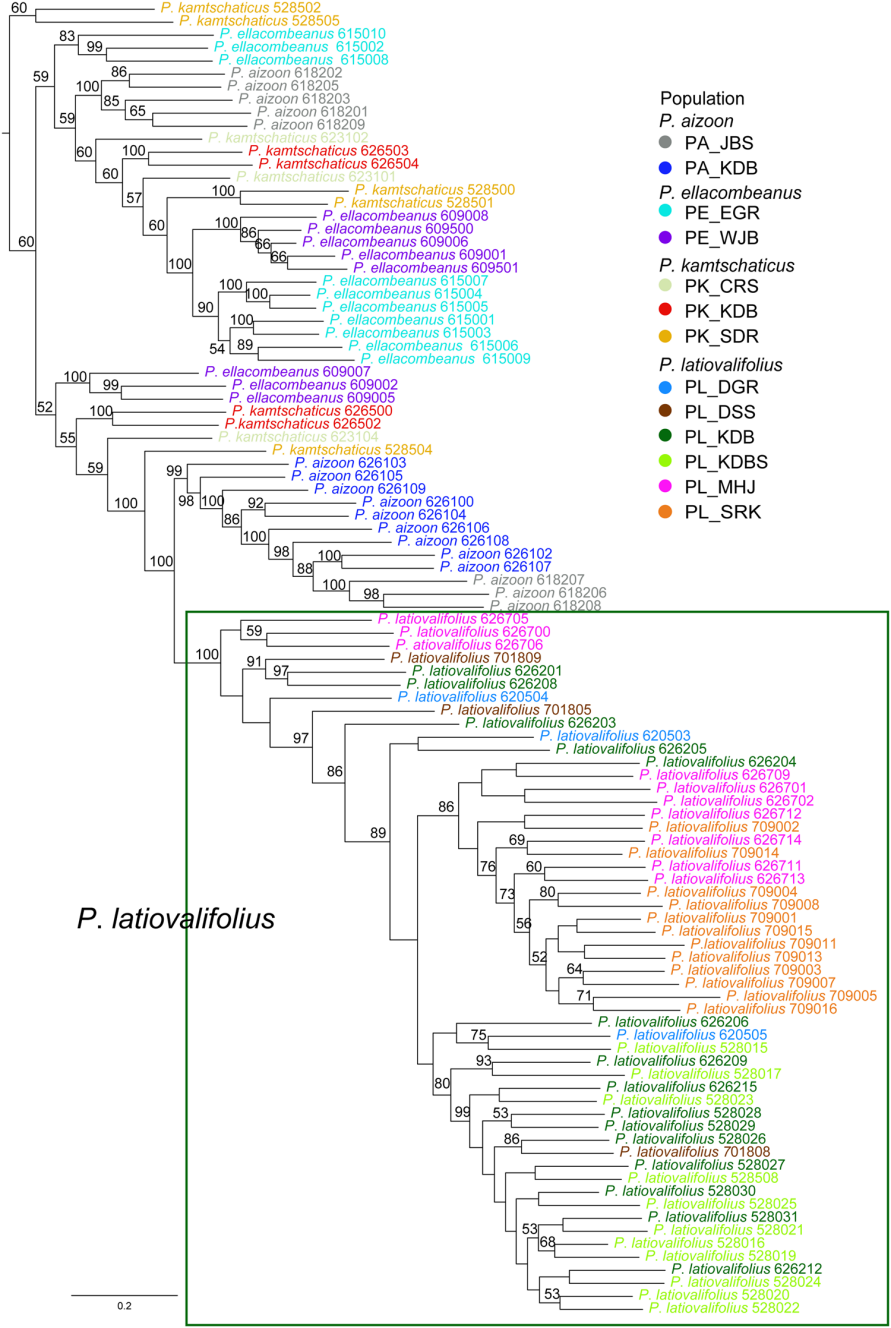
Maximum likelihood (ML) tree produced by IQ-TREE and visualized by the Interactive Tree of Life (iTOL) based on the GBS-derived 6642 SNPs for 101 accessions of *P*. *latiovalifolius* and sympatric *Phedimus* species. Numbers on branches are bootstrap support (BS) values of > 50% with 1000 bootstrap replicates.

**Figure 4:**
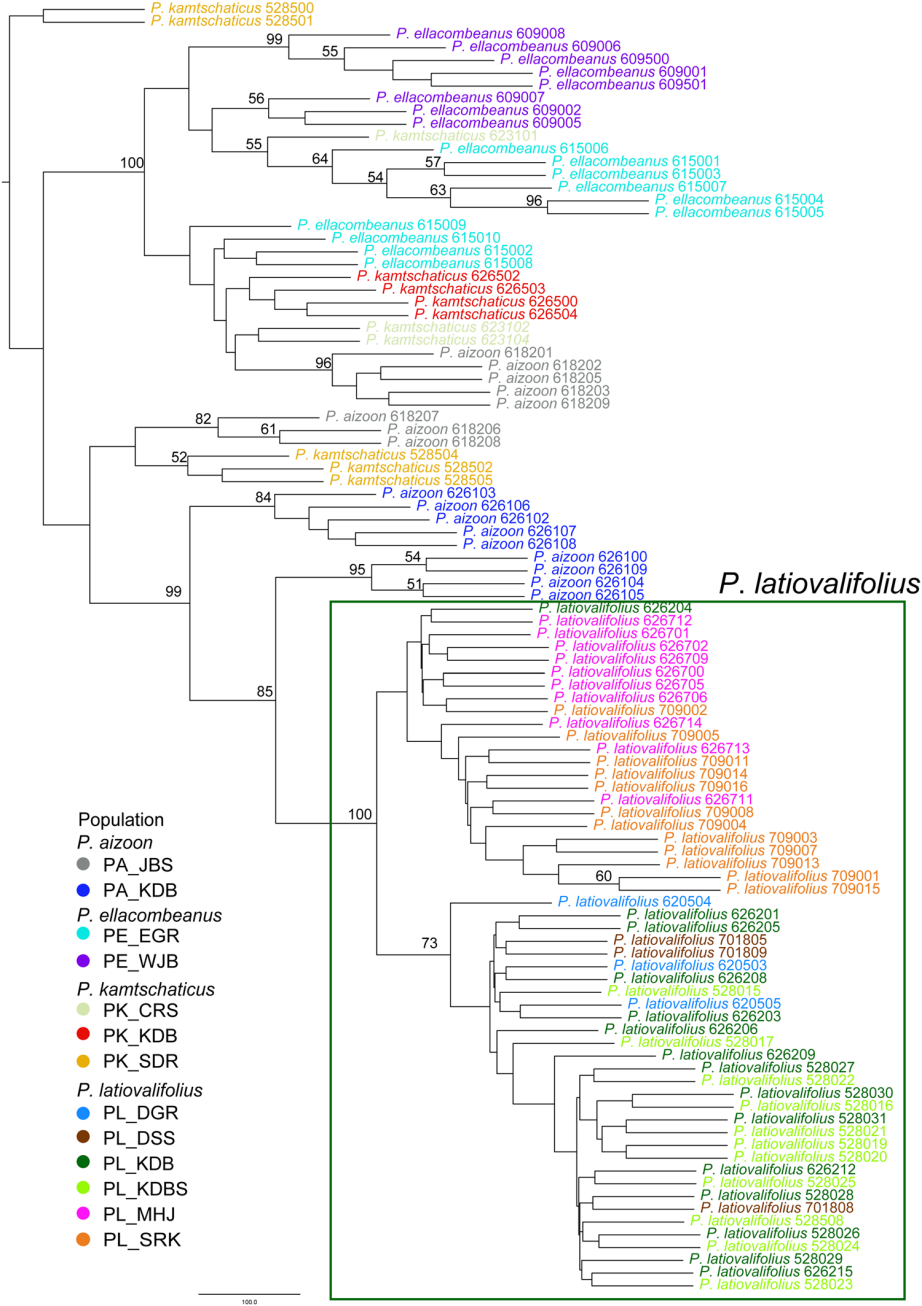
SVDQuartets bootstrap consensus tree generated PAUP and visualized by the Interactive Tree of Life (iTOL) based on the GBS-derived 6642 SNPs for 101 accessions of *P*. *latiovalifolius* and sympatric *Phedimus* species. Numbers on branches are bootstrap support (BS) values of > 50% with 100 bootstrap replicates.

Phylogenetic analyses were conducted based on 6642 SNPs obtained from GBS analysis to elucidate the relationships among 101 accessions of *P. latiovalifolius* and other *Phedimus* species. The analyses employed ML IQ-TREE (Fig. 3) and SVDQuartets (Fig. 4) trees. *P. latiovalifolius* emerged as the sole monophyletic species with robust bootstrap support (100% BS for each tree), while the species identified as *P. aizoon, P. kamtschaticus*, and *P. ellacombeanus* were not monophyletic. Both ML and SVDQuartets trees indicated *P. aizoon* as the sister species to *P. latiovalifolius*, although *P. aizoon* was not monophyletic. In the ML tree, the paraphyletic Geumdaebong Peak population (PA_KDB), inclusive of the three accessions of PA_JBS, was identified as the sister to *P. latiovalifolius* (100% BS). The SVDQuartets tree suggested a similar relationship with 99% BS. Further examination within *P. latiovalifolius* revealed, albeit weakly supported, two major lineages in both phylogenetic trees (except several accessions placed outside of two major lineages on ML tree). The first lineage (BS < 50% on ML and 73% BS on SVDQuartets trees) primarily comprised the Geumdae-bong Peak populations (PL_KDB and PL_KDBS). The second lineage included the Mt. Seorak population, coupled with the Manhang-jae Pass population (86% BS on the ML tree and BS < 50% on the SVDQuartets tree). The SVDQuartets tree (Fig. 4) suggested that the Mt. Dosol (PL_DSS) and Daegwallyeong Pass (PL_DGR) populations were embedded within the clade of PL_KDB and PL_KDBS.

### Genetic diversity and analysis of molecular variation

The genetic diversity of *P. latiovalifolius*, excluding two small populations (sample size of ≤ 3), was assessed using STACKS (Table 2). Expected heterozygosity ranged from 0.2073 (PL_MHJ) to 0.2309 (PL_KDBS), with a mean of 0.2190. Nucleotide diversity (π) varied, with the PL_SRK population displaying the highest (0.2397) and PL_MHJ the lowest (0.2192), averaging at 0.2296. For comparative analysis with congeneric species, two populations of *P. aizoon* from Gangwon-do Province exhibited the lowest mean expected heterozygosity (0.1783) and π (0.1906). Conversely, two populations of *P. ellacombeanus* from the southern Korean Peninsula displayed the highest expected heterozygosity (0.2214) and nucleotide diversity (0.2351).

**Table 2:** Molecular diversity indices estimated in the populations of *P. latiovalifolius* and congeneric *Phedimus* species.

Species	Pop ID	N/I	Num Indv	Pri-vate	Polymorphic Sites (%)	P	Obs Het	Exp Het	π	Fis
*P. aizoon*	PA_JBS	8	7.5548	1	3664(58)	0.8609	0.1947	0.1902	0.2038	0.0232
	PA_KDB	9	8.1974	1	3350(53)	0.8803	0.1624	0.1663	0.1773	0.0405
	Mean		7.8761		3507(55.5)	0.8706	0.17855	0.1783	0.1906	0.03185
*P. ellacombeanus*	PE_WJB	8	7.6522	6	3253 (51.4)	0.8276	0.3136	0.2141	0.2291	− 0.1706
	PE_EGR	10	9.7791	19	3990 (63.1)	0.8227	0.3282	0.2287	0.2411	− 0.1812
	Mean		8.7157		3621(57.3)	0.8252	0.3209	0.2214	0.2351	− 0.1759
*P. latiovalifolius*	PL_KDB	15	8.2141	0	3829 (60.1)	0.8441	0.2253	0.208	0.2216	− 0.0028
	PL_KDBS	11	16.8433	19	3739 (59.1)	0.8179	0.3328	0.2309	0.238	− 0.2028
	PL_MHJ	10	9.2701	0	3700(58.5)	0.8448	0.221	0.2073	0.2192	0.0003
	PL_SRK	12	11.8777	0	3741 (59.2)	0.8212	0.3288	0.2296	0.2397	− 0.1891
	Mean		11.5513		3752(59.3)	0.832	0.2770	0.2190	0.2296	− 0.0986

Pop ID: Population code (See Table 1 for population abbreviation), N/I: Number of individuals sampled in this population at this site, Num Indv: Mean number of individuals per locus, Private: Number of private alleles, P: Mean frequency of the most frequent allele at each locus, Obs Het: Mean observed heterozygosity (The proportion of individuals that are heterozygotes), Exp Het: Mean expected heterozygosity under Hardy–Weinberg equilibrium, π: Mean value of π (An estimate of nucleotide diversity), Fis: Mean measure of FIS (value of inbreeding coefficient) in this population.

Within the mountainous endemic species, *P. latiovalifolius*, majority of the variation (86%) was observed within populations, while the remaining variation (14%) was distributed among populations (Table 3). Similar levels of genetic variation were found in other *Phedimus* species; *P. aizoon* (within-population variation of 80.4%, among population variation of 19.6%), and *P. ellacombeanus* (80.2%, 19.8%). Across the three species, variations among species, among populations, and within populations were 20%, 20.7%, and 59.3%, respectively. Pairwise genetic differentiation (*F*_st_) was notable between all pairs of *P. latiovalifolius* and other *Phedimus* species, ranging from 0.151 (PL_MHJ and PA_KDB) to 0.286 (PL_KDBS and PE_WJB) (Table 4). Within *P. latiovalifolius* populations, pairwise genetic differentiation ranged from 0.012 (PL_SRK and PL_MHJ) to 0.075 (PL_MHJ and PL_KDBS). Overall, *P. aizoon* and *P. ellacombeanus* populations showed comparable levels of intraspecific genetic differentiation (0.096 and 0.065, respectively). Considering both intra- and interspecific genetic differentiation, it is apparent that all populations of *P. latiovalifolius* were genetically differentiated from congeneric species.

**Table 3:** Analysis of molecular variance (AMOVA) results for genetic variation found within and among populations and species of *P. latiovalifolius* and other sympatric *Phedimus* species.

Source of Variation	SSD	d.f	MS	Var-comp	%Var
*P. aizoon* 2 pop (17 indv.) [Rho_st = 0.196, P-value 0.001]					
Within Populations	8808.315	15	587.221	587.221	80.4
Among populations	1797.154	1	1797.154	142.839	19.6
*P. ellacombeanus* 2 pop (18 indv.) [Rho_st = 0.198, P-value 0.001]					
Within populations	6772.489	16	423.281	423.281	80.2
Among populations	1353.914	1	1353.914	104.696	19.8
*P. latiovalifolius* 4 pop (48 indv.) [Rho_st = 0.14, P-value 0.001]					
Within populations	22,346.19	44	507.868	507.868	86
Among populations	4443.367	3	1481.122	82.928	14
*Phedimus* 3 species 8 pop (83 indv.) [Rho_st = 0.407, Rho_sc = 0.259, P-value 0.001, Rho_ct = 0.2, P-value 0.012]					
Within Populations	37,926.99	75	505.693	505.693	59.3
Among populations	7594.435	5	1518.887	176.478	20.7
Among species	16,796.55	2	8398.274	170.638	20

SSD = Sum of squares, d.f. = Degree of freedom, MS = Mean squares, Var-comp = Variance components, %VAR = percentage of total variance.

**Table 4:** Pairwise genetic differentiation based on *F*_st_ (Phi_st_) between all populations of *P. latiovalifolius* and congeneric *Phedimus* species.

Species	Pop ID	PA_JBS	PA_KDB	PE_WJB	PE_EGR	PL_KDB	PL_KDBS	PL_MHJ	PL_SRK
*P. aizoon*	PA_JBS	–	0.001	0.001	0.001	0.001	0.001	0.001	0.001
	PA_KDB	0.096	–	0.002	0.001	0.001	0.001	0.001	0.001
*P. ellacombeanus*	PE_WJB	0.085	0.181	–	0.001	0.001	0.001	0.001	0.001
	PE_EGR	0.052	0.142	0.065	–	0.001	0.001	0.001	0.001
*P. latiovalifolius*	PL_KDB	0.214	0.171	0.274	0.243	–	0.001	0.001	0.001
	PL_KDBS	0.234	0.192	0.286	0.258	0.013	–	0.001	0.001
	PL_MHJ	0.2	0.151	0.262	0.229	0.049	0.075	–	0.001
	PL_SRK	0.215	0.168	0.271	0.24	0.061	0.074	0.012	–

### Population structure and gene flow

**Figure 5:**
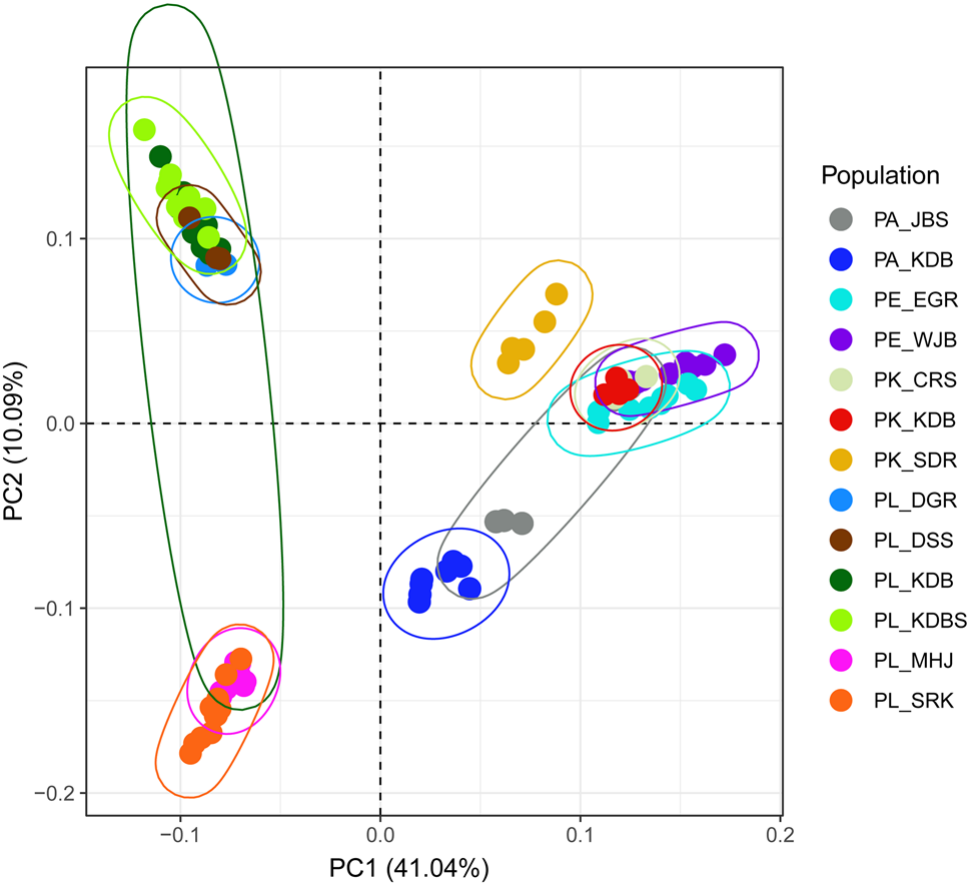
Score plot for principal component analysis (PCA) for 101 accessions of *P*. *latiovalifolius* and sympatric *Phedimus* species based on the GBS-derived 6642 SNPs.

**Figure 6:**
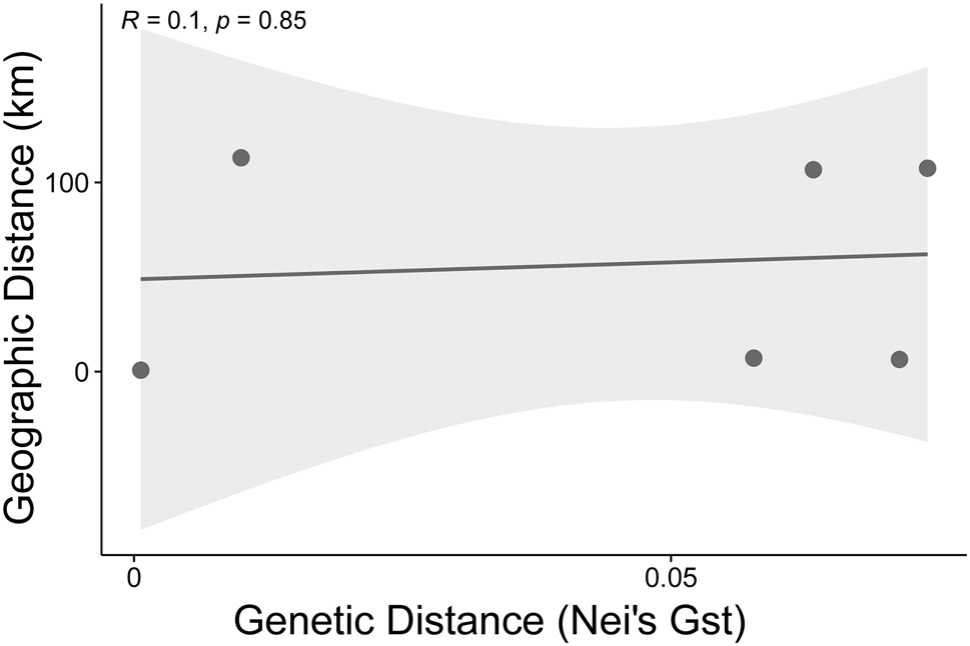
Relationship between geographic distance (km) and genetic distances (Nei’s *G*st) among the populations of *P. latiovalifolius.*

**Figure 7:**
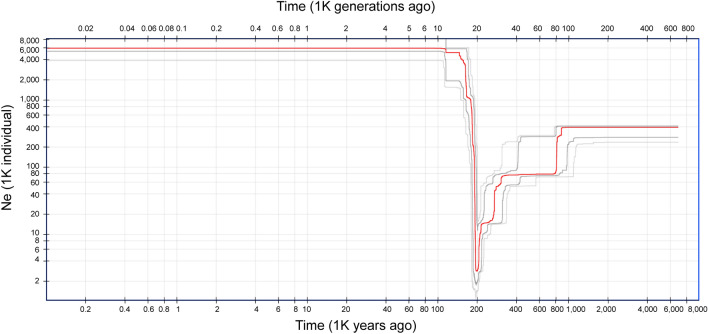
Inferred demographic history of *P. latiovalifolius* based on 54 individuals. Orange line: median of 200 inferences based on subsampling. Dark grey lines: 12.5% and 87.5% confidence interval of the inference. Light grey lines: 2.5% and 97.5% confidence interval of the inference.

The PCA results based on 6642 SNP loci elucidated the genetic relationships within *P. latiovalifolius* populations and their connections with closely related *Phedimus* species (Fig. 5). *P. latiovalifolius* exhibited distinctiveness from all other congeneric species, forming an independent cluster on PCA plot. On the other hand, all the other congeneric species were overlapped and clustered together. Within *P. latiovalifolius*, two discernible genetic clusters emerged. The first cluster comprised populations from Geumdaebong Peak South (PL_KDBS), Geumdaebong (PL_KDB), Mt. Dosol (PL_DSS), and Daegwanryong Pass (PL_DGR), excluding one accession of PL_KDB. The second cluster encompassed two geographically separate populations: Mt. Seorak (PL_SRK) and Manhangjae Pass (PL_MHJ). For *P. aizoon*, two populations (excluding several accessions of PA_JBS) appeared isolated from other congeneric species, namely *P. kamtschaticus*, *P. ellacombeanus*, and *P. latiovalifolius*. All but one population (PK_SDR) of *P. kamtschaticus*, and the geographically separated coastal *P. ellacombeanus*, grouped together. The northernmost population of *P. kamtschaticus* (PK_SDR) exhibited some distance from the *P. kamtschaticus* and *P. ellacombeanus* clusters. The Mantel test revealed no significant correlations between geographic and genetic distances for the six populations of *P. latifovalifolius* (r = 0.1, p = 0.85) (Fig. 6). The demographic history of *P. latifovalifolius* inferred from folded SNP frequency spectra (SFS) indicated a bottleneck in its effective population size between 100 and 1000 thousand years ago (kya) based on 95% confidence interval, with a bottom approximately 200 kya (Fig. 7). The effective populations of *P. latifovalifolius* appeared as maintained quite stable since a recovery from the bottleneck, approx. 100 kya.

**Figure 8:**
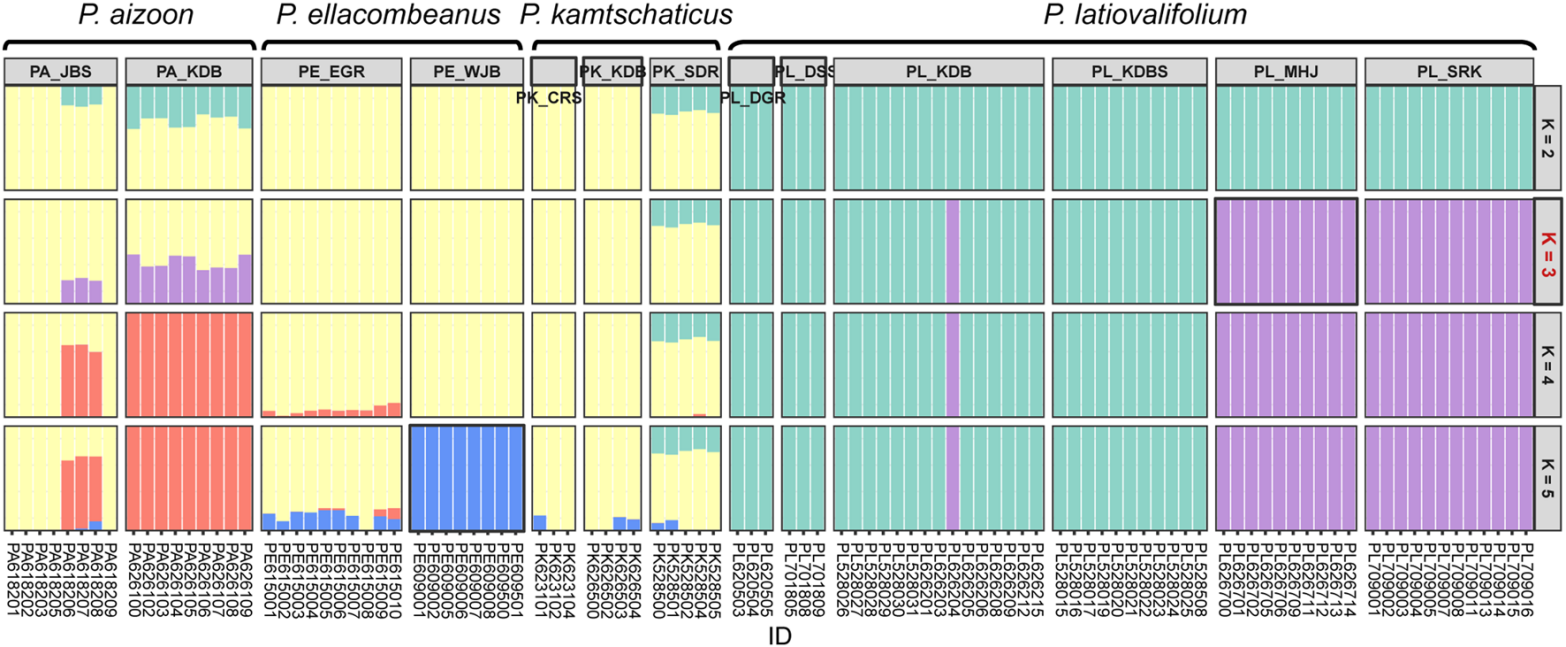
Stacked bar plots of Q estimates of ancestry fractions using the best K (3) and comparative K (2, 4, and 5) values based on the GBS-derived SNPs.

The population genetic structure was assessed by individual ancestries of 101 accessions of *P. latiovalifolius* (six populations) and closely related *Phedimus* species (two populations of *P. aizoon*, two of *P. ellacombeanus*, and three of *P. kamtschaticus*) using ADMIXTURE V1.3.0^38^. Although the optimal K value was determined as K = 3, reflecting the lowest cross-validation error (Supplementary Fig. S1), bar plots of the Q estimates for K = 2, 3, 4, and 5 are presented for comparison (Fig. 8). The partitioned bar plots for all K values reveal distinct non-admixture genetic assignment patterns for *P. latiovalifolius* populations, while the populations of all other *Phedimus* species shared the same or admixed genetic clusters in common. Within *P. latiovalifolius*, two genetic clusters were identified at K = 3, aligning with ML and SVDQuartet trees (Figs. 3 and 4) and PCA results (Fig. 5): one comprising two geographically separated populations (PL_MHJ and PL_SRK), and the other including the remaining populations (PL_DGR, PL_DSS, PL_KDB, and PL_KDBS) except one accession from PL_KDB (PL626204). At K = 3, *P. ellacombeanus*, two populations of *P. kamtschaticus* (PK_CRS and PK_KDB), and several accessions of *P. aizoon* (PA_JBS) shared the same non-admixture genetic assignment. *P. aizoon* (all PA_KDB accessions and three PA_JBS accessions) and *P. kamtschaticus* (all PK_SDR accessions) were identified as admixtures of the two genetic clusters. Genetic structure analysis did not differentiate *P. kamtschaticus* from *P. aizoon* or *P. ellacombeanus*.

**Figure 9:**
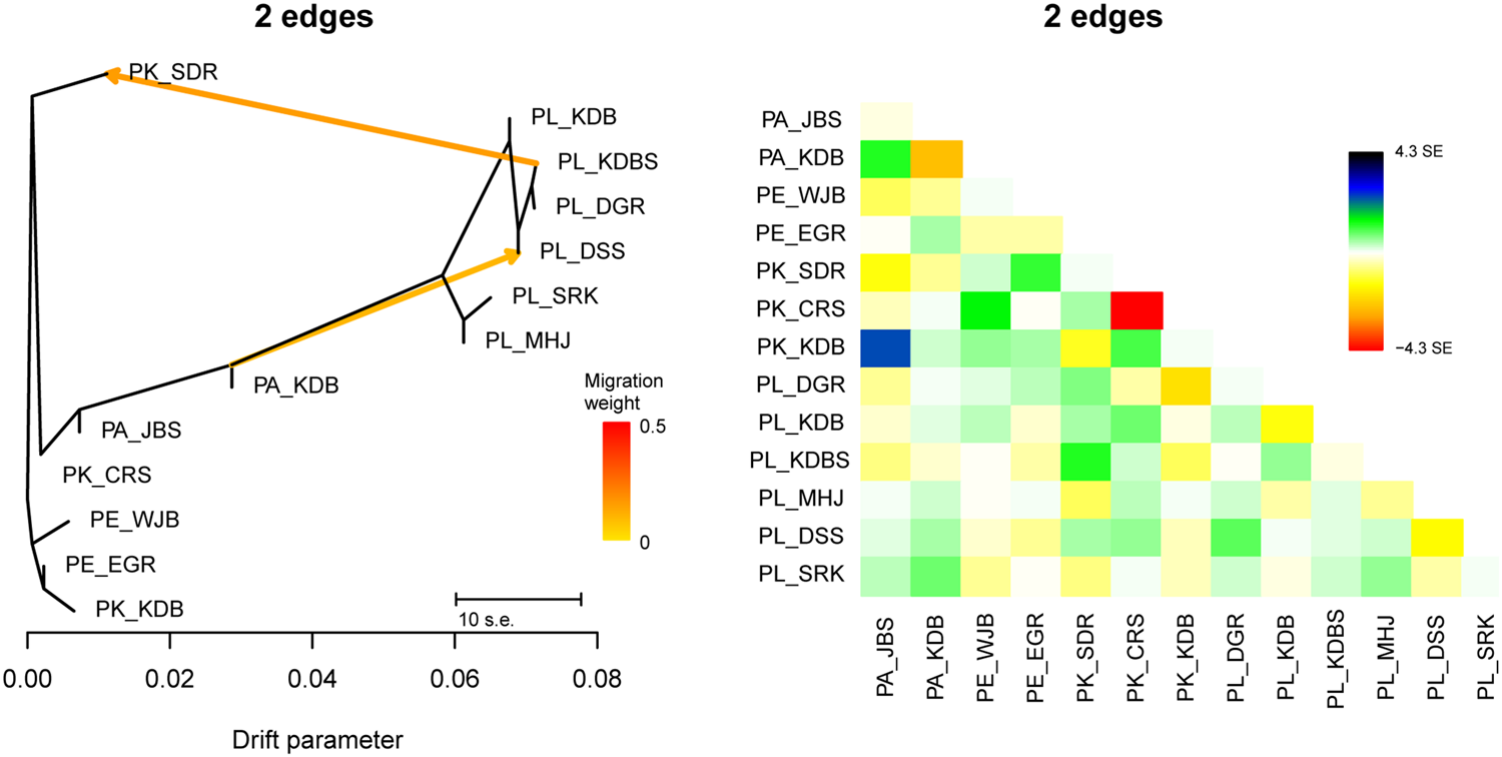
**A** Maximum-likelihood (ML) tree with optimal two migration events (M2) inferred with TreeMix. The scale bar shows 10 times the average standard error of entries in the sample covariance matrix. Two estimated migration events are represented by an arrow and are colored according to their migration weight. **B** Residual fit plotted from the ML tree in (**A**). Residuals above zero represent populations that are more closely related to each other and thus are candidates for admixture events.

The best TreeMix graph was chosen as M2 (two migration edges) with the highest value for Δ*m* (the second-order rate of change in likelihood across values of *m*) in optM, explaining 99.46% variances (Supplementary Fig. S2). The threshold (99.8%) recommended by Pickrell and Pritchard^39^ for the proportion of explained variance was not achieved for all tested migration events from 0 to 10. The M2 TreeMix graph displayed a topology similar as a species tree (SVDQuartet tree; Fig. 4); i.e., the monophyly of *P. latiovalifolius* with two major lineages, a close relationship between *P. latiovalifolius* and *P. aizoon* (specifically PA_KDB population), and that the remaining species of *P. aizoon*, *P. kamtschaticus*, and *P. ellacombeanus* were intermixed (Fig. 9). Treemix results indicated two gene flow events between *P. latiovalifolius* and either *P. aizoon* (PA_KDB population) or *P. kamtschaticus* (PK_SDR population), corresponding to the population genetic structure analyzed by ADMIXTURE (Fig. 8). The direction of gene flow was PL_KDBS of *P. latiovalifolius* into PK_SDR of *P. kamtschaticus* and PA_KDB of *P. aizoon* into PL_DSS of *P. latiovalifolius*, which did not support the hypothesis of hybrid origin of *P. latiovalifolius* between *P. aizoon* and *P. kamtschaticus*. Furthermore, we found no indication of any gene flow events, consistent with the hybrid origin hypothesis, either when the number of migration edges in the model increased (Supplementary Fig. S3) or when two migration edges were iterated for ten replicates (Supplementary Fig. S4). None of the possible three- and four-population tests (*f*_3_ statistics/*f*_4_ statistics) implemented in TreeMix supported gene flow between *P. aizoon* and *P. kamtschaticus* involving the hybrid origin of *P. latiovalifolius*. All significantly negative *f*_3_ statistics (Z-score <  − 3) as well as negative or positive* f*_4_ statistics (Significant Z-score >|3|) did not infer that *P. latiovalifolius* was the product of admixture between hypothesized parents in its hybrid origin (Supplementary Tables S1, S2, and S3).

## Discussion

### Origin, genetic diversity, and population structure of *P. latiovalifolius*

Based on the GBS-derived genome-wide SNPs, this study successfully confirmed the monophyly of *P. latiovalifolius* and determined its sister lineage using concatenation-based ML and coalescent-based SVDQuartets analyses. All 54 accessions of *P. latiovalifolius* from the six populations (PL_DSS, PL_DGR, PL_MHJ, PL_SRK, PL_KDB, and PL_KDBS) formed a 100% BS-supported clade in both phylogenetic trees (Figs. 3 and 4). Initially, *P. latiovalifolius* was described as a distinct *Phedimus* species based on morphological differences in the broadly ovate leaves of the terminal stem arranged in a rosette form, as well as its restricted distribution to Geumdaebong Peak, Mount Taebaek, in Gangwon-do Province. Such morphological and geographical distinctions are now supported by molecular evidence based on its monophyly and genetic differentiation from three representative congeneric species (*P. aizoon*, *P. kamtschaticus*, and *P. ellacombeanus*). Genetic differentiation of *P. latiovalifolius* was further corroborated unambiguously by genetic structure and PCA analyses (Fig. 5 and 8), as well as pairwise genetic differentiation based on *F*_st_ (Table 4). *P. latiovalifolius* demonstrated the non-admixture genetic assignment patterns of exclusive genetic clusters distinct from other *Phedimus* species on ADMIXTURE bar plot. PCA score plot also displayed an independent placement of *P. latiovalifolius* apart from the intermixed cluster of other congeneric species. Thus, this study further confirmed the monophyly of *P. latiovalifolius*, which was preliminarily suggested based on the limited chloroplast and nrDNA ITS noncoding sequences^26,27^.

In terms of phylogenetic relationships among closely related *Phedimus* species, the monophyletic *P. latiovalifolius* shared the most recent common ancestor with *P. aizoon* in both ML and SVD Quartets trees, although *P. aizoon* was not monophyletic. Particularly, *P. latiovalifolius* was closest to the Geumdaebong Peak population of *P. aizoon* (PA_KDB), the type locality of *P. latifolvalifolius*, in the SVD Quartets tree (Fig. 4; 99% BS support). In the ML tree (Fig. 3; 100% BS support), it was also closest to Geumdaebong Peak population (PA_KDB), but inclusive few accessions of the Mt. Jangbyeong (PA_JBS) population of *P. aizoon*. The nrDNA ITS tree, based on broad sampling, identified several distinct geographical lineages of *P. aizoon*, suggesting that one geographical lineage in the Korean Peninsula might have been involved in the origin of *P. latifovalifolius* during the Late Miocene^26,27^. *P. aizoon* is a highly polymorphic species with extensive polyploid and aneuploid series that often intergrades with the sympatric, highly variable *P. kamtschaticus*^11,13^. The ploidy level of *P. latiovalifolius* and the specific geographical lineage of *P. aizoon* that contributes to *P. latiovalifolius* require further investigation based on broad sampling to fully understand the origin of the high mountain endemic to the Korean Peninsula.

The current results do not support the hybrid origin of *P. latiovalifolius* between *P. aizoon* and *P. ellacombeanus* or between *P. kamtschaticus* and *P. ellacombeanus*^20^, corroborating the previous findings based on morphological and allozyme data^24^. The M2 TreeMix graph displayed two potential gene flow events between *P. latiovalifolius* and either *P. aizoon* (PA_KDB population) or *P. kamtschaticus* (PK_SDR population) (Fig. 9). The direction of gene flow was PL_KDBS of *P. latiovalifolius* into PK_SDR of *P. kamtschaticus* and PA_KDB of *P. aizoon* into PL_DSS of *P. latiovalifolius* without any indication of gene flow events consistent with the hybrid origin hypothesis, either when the number of migration edges in the model increased (Supplementary Fig. S3) or when two migration edges were iterated for ten replicates (Supplementary Fig. S4). None of the three- and four-population tests (*f*_3_ statistics/*f*_4_ statistics) implemented in TreeMix inferred that *P. latiovalifolius* was the product of admixture involving the hybrid origin of *P. latiovalifolius* (Supplementary Tables S1, S2, and S3).

### Korean Baedudaegan Mountains and a glacial refugium for boreal and temperate species

*Phedimus latiovalifolius*, now confirmed as a Korean endemic species, occurs disjunctively in narrow ranges of several adjacent high mountains (within the main mountain range and watershed crest line known as the Baekdudaegan, 'BDDG') in the east-central Korean Peninsula. The BDDG is a well-known biodiversity hotspot, harboring high species richness and endemism. Several phylogeographic and population genetic studies have suggested that the BDDG serves as a biodiversity reservoir, and this is associated with its role as a glacial refugium, presenting relevant genetic evidence from several plant species found in the BDDG area^18,40–44^. In the case of a large assemblage of boreal and temperate plants, it has been suggested that the BDDG should be regarded as glacial refugia in addition to other well-known East Asian Pleistocene refugia (e.g., the Hengduan Mountains, the Nanling Mountains, or the central China Mountains) ^45–47^.

Chung et al. (2017)^41^ conducted a review of the genetic evidence supporting the role of the BDDG as a glacial refugium for several Korean species (e.g., *Kalopanax* *septemlobus* Koidz.^48^, *Pinus koraiensis* Siebold & Zucc.^49^, *Quercus* *variabilis* Blume^50^, *Veratrum album* ssp. *oxysepalum* (Turcz.) Hultén^51^, and 16 other species^40^). These studies generally revealed the expected genetic pattern, with plants persisting in refugia, consistent with the "southern richness to northern purity" paradigm^52^ and the basic Expansion–Contraction model (EC model) of Quaternary demography^53^ explaining the spatial genetic consequences with lower genetic diversity in postglacially colonized northern regions comparing to southern glacial refugia throughout the repeated cycles of latitudinal range shifts. They also exhibited relatively high levels of intrapopulation genetic diversity, ancestral haplotypes, and/or significant numbers of unique haplotypes/alleles. Paleoecological data further supported these genetic studies, indicating that the BDDG sustained a mixture of boreal and temperate forests during the Last Glacial Maximum (LGM).

*Phedimus latiovalifolius* is distributed disjunctively on several adjacent high mountains in the central BDDG. Given its geographically disjunctive distributional pattern and the potential role of the BDDG as an East Asian Pleistocene refugium, *P. latiovalifolius* could be considered as a rare, endemic species that persisted in the BDDG refugium through altitudinal shifts throughout glacial/interglacial cycles. The levels of genetic diversity and differentiation in *P. latiovalifolius* can be compared to those of other plant species mainly occurring in the BDDG^41^. Although a direct comparison between different types of molecular data based on allozymes^41^ and genome-wide SNPs by GBS (current study) is not feasible, it seems that the herbaceous perennial *P. latiovalifolius* (mean H_e_ = 0.219) exhibits higher genetic diversity than endemic plants (mean H_e_ = 0.063), narrowly distributed plants (mean H_e_ = 0.105), and short-lived herbaceous perennials (mean H_e_ = 0.096). An allozyme study based on nine populations of *P. latiovalifolius* sampled from its type locality (Geumdaebong Peak) found a similar level of mean heterozygosity (H_e_ = 0.26)^24^ as the current GBS-based study. Additionally, the same study suggested that narrowly distributed *P. latiovalifolius* in the BDDG contained a similar level of genetic diversity as more widely distributed and sampled congeners, such as *P. aizoon* (H_e_ = 0.255) and *P. kamtschaticus* (H_e_ = 0.257) (see also H_e_ = 0.203 for *P. kamtschaticus* in earlier Chung et al.’s study^54^). Therefore, the high genetic diversity of *P. latiovalifolius* appears to align with the general pattern found in other BDDG plant species. However, the population differentiation measured by *F*_st_ for *P. latiovalifolius* (mean *F*_st_ = 0.047) suggests much lower differentiation than that of narrowly distributed (*F*_st_ or *G*_st_ = 0.242) or short-lived herbaceous perennials (*F*_st_ or *G*_st_ = 0.233). Thus, the very low population differentiation found in *P. latiovalifolius* is exceptional for plant species in the BDDG, which typically show low to moderate among-population genetic variability (mean *F*_st_ = 0.175). However, this statement might be somewhat misleading, as *F*_st_ and *G*_st_ range widely, from 0.027 (herbaceous perennial *Adenophora grandiflora* Nakai; Campanulaceae) to 0.627 (herbaceous perennial *Leontice microrryncha* S.Moore; Berberidaceae) (see Table 1 of Chung et al., 2017^40,41^). Genetic differentiation greatly depends on breeding systems, pollination and dispersal modes, demographic history, ecological attributes, and many other factors^55–57^. Given the short dispersal distances of seeds via splash rain seed dispersal mechanisms typically found in the genus and family^58–60^, the low level of population differentiation in *P. latiovalifolius* was unexpected and contrasted with *P. takesimensis* on Ulleung Island^26^. We hypothesize that the two geographically disjunct patterns among populations in *P. latiovalifolius*, corroborated by PCA and genetic structure analyses, could be formed by postglacial contraction (i.e., vicariance resulting from climate-related population fragmentation and local extinction during interglacial periods) rather than by long-distance dispersal. The overall genetic patterns of plant species restricted to the BDDG can be further synthesized based on comparative phylogeographic and population genetic studies, including the diverse life history traits of plant species.

Given its levels of genetic diversity in comparison to more widely occurring congeneric species, *P. latiovalifolius* appears less likely to go extinct due to genetic factors for conservation perspectives. Furthermore, following a demographic expansion that began 100,000 years ago, the estimated effective population size (*Ne*) change revealed a stable size for *P. latiovalifolius* (Fig. 7). However, the abundance of P*. latiovalifolius* along both sides of hiking trails may negatively affect pollinator visitation and harm plants that support species establishment and growth. In addition, hiking trails may promote the introduction of non-native plant species into natural areas, thereby facilitating competition with *P. latiovalifolius* and other native plant species. The over-harvesting of this succulent species with high ornamental values in rock gardens and succulent gardens could be an additional threat. The long-term survival of *P. latiovalifolius* may be negatively impacted by the ongoing climate change, which is expected to result in an upward shifting of the species due to rising temperatures and altered precipitation patterns. For the conservation of *P. latiovalifolius*, we suggest securing seeds or fragments of plants from two geographically disjunct groups of populations as ex situ strategies. In addition, it is recommended to conduct demographic monitoring of current populations concurrently with ex situ conservation strategies.

In conclusion, we determined the taxonomic identity of *P. latiovalifolius* by providing extensive genetic evidence for its monophyly based on the genome-wide SNPs derived by GBS. We conducted a large-scale population genetic and phylogenetic analyses of six populations of *P. latiovalifolius* and compared them to representative populations of other sympatric *Phedimus* species: *P. kamtschaticus*, *P. aizoon*, and *P. ellacombeanus*. The monophyly of *P. latiovalifolius* was strongly demonstrated, and its genetic distinctiveness was further unambiguously corroborated by genetic structure, PCA, and genetic differentiation analyses. *P. latiovalifolius* has been confirmed as a Korean-endemic species that occurs disjunctively in several adjacent high-altitude mountains located within the BDDG on the Korean Peninsula. It can be regarded as a rare endemic species that has persisted in the BDDG refugium throughout glacial/interglacial cycles through altitudinal shifts. Furthermore, the populations of *P. latiovalifolius* showed a geographically disjunct pattern, presumably formed by post-glacial range contraction rather than by recent long-distance dispersal. Regarding the origin of *P. latiovalifolius*, the phylogenetic relationships among *Phedimus* species did not reveal any genetic evidence for the potential hybrid hypothesis previously proposed. Although it displayed genetic closeness to *P. aizoon*, *P. latiovalifolius* was not phylogenetically closely related to its purported parent species, without any indication of the admixture product or any gene flow events responsible for its hybrid origin.

## Materials and methods

### Plant materials

To detect GBS-derived genome-wide SNPs, we sampled 145 wild accessions of *Phedimus latiovalifolius* (85 accessions)*, P. aizoon* (20), *P. ellacombeanus* (20), and *P. kamtschaticus* (20) (Fig. 2 and Table 1). *P. latiovalifolius* was collected from six populations of Daegwallyeong Pass (population code PL_DGR; 952 m), Geumdaebong Peak, Mt. Taebaek (PL_KDB; 1345 m) (Fig. 1 A-C), the south from Geumdaebong Peak, Mt. Taebaek (PL_KDBS; 1311 m), Manhangjae Pass, Mt. Hambaek (PL_MHJ; 1297 m), Mt. Dosol (PL_DSS; 1029 m) (Fig. 1 D), and Mt. Seorak (PL_SRK; 1010 m**)** (Fig. 1E,F) in mountainous areas of central Korea. These six populations represented the entire range of *P. latiovalifolius* populations in South Korea. Two sympatric species, *P. aizoon* and *P. kamtschaticus* were collected from the overlapped distribution areas with *P. latiovalifolius*; two populations of *P. aizoon* from Mt. Jangbyeong (PA_JBS; 703 m) and Geumdaebong Peak, Mt. Taebaek (PA_KDB; 1345 m), and three populations of *P. kamtschaticus* from Sottongnyeong (PK_SDR; 98 m), Mt. Cheongryang (PK_CRS; 200 m), and Geumdaebong Peak, Mt. Taebaek (PK_KDB; 1345 m). Two populations of *P. ellacombeanus* were collected from Wujebong Peak (PE_WJB; 92 m) and Egari (PE_EGR; 21 m) on the southern seashore area of the Korean Peninsula. As *Phedimus* species are neither highly threatened nor legally protected, permits were not required to be collected. All samples were collected from wild populations on the Korean Peninsula and one representative voucher specimen per each population were deposited at the Ha Eun Herbarium (SKK), Sungkyunkwan University, Korea (see the voucher information in Table 1). Experimental research and field studies on wild plants, including the collection of plant materials, complied Sungkyunkwan University and Korean guidelines and legislation. Species identification was performed by Seung-Chul Kim, an expert in Korean *Phedimus* species. In this study, we complied with the IUCN Policy Statement on Research Involving Species at Risk of Extinction and the Convention on the Trade in Endangered Species of Wild Fauna and Flora.

### DNA isolation, GBS Library construction, sequencing, and variants calling

Total genomic DNA was extracted from silica gel-dried leaves using a DNeasy Plant Mini kit (Qiagen, Valencia, CA, United States) following the manufacturer’s instructions. The extracted DNAs were submitted to Seeders Inc. (Daejeon, Korea) for GBS library construction. Libraries were prepared by the restriction enzyme digestion of DNA with ApeK1 (GCWGC), followed by ligation of barcoded adapters according to a protocol modified by Elshire et al.^61^. The pooled libraries, each containing either 96 or 49 samples, were sequenced on an Illumina HiSeq X system (Illumina, San Diego, CA, USA) with an average 151 bp length for paired-end reads. Two sequencing runs were conducted consecutively. Sequence data were deposited in the NCBI Sequence Read Archive (SRA BioProject number PRJNA1037601, https://www.ncbi.nlm.nih.gov/bioproject/PRJNA1037601).

Raw reads were demultiplexed using barcode sequences associated with each sample to generate separate FASTQ files for all 145 samples. Adapter sequences were removed using Cutadapt v1.8.3^62^, and the demultiplexed reads were trimmed using DynamicTrim (phred score ≥ 20) and LengthSort (short read length ≥ 25 bp) in SolexaQA v1.13^63^. Poor-quality sequences with Phred quality scores below Q = 20 (or an error probability of 0.05) were removed, and short read lengths of < 25 bases were discarded.

Variant calling was conducted by the DeNovoGBS function implemented in NGSEPcore_4.1.0^64^. Default parameters were used to discover and genotype Single Nucleotide Variants (SNVs): K-mer length 31, the maximum number of read clusters 2,000,000, maximum value allowed for a base quality score, 30; and minimum variant quality, 40. The obtained SNVs were filtered using VCFTOOLS v0.1.16^65^; the applied parameters were mac (Minor Allele Count) 3, min-meanDP (minimum mean depth values) 3, minQ (minimum Quality value) 30, max-missing (maximum missing genotypes) 0.5, hwe (sites for Hardy–Weinberg Equilibrium) 0.01, and min-r2 (minimum squared correlation coefficient between genotypes) 0.8. A total of 44 samples with extensive missing data (> 50%) were removed to maintain optimal dataset quality. The SNP data matrix, including 6642 SNPs shared by all 13 populations (-r 0.75 and -p 13 applied), was generated by the program ‘populations’ in STACKS v1.48^66^ in multiple output formats of variant call format (VCF), STRUCTURE, and Phylip. PLINK v1.90b6.21^67^ was used to convert the SNP data into binary PLINK (.bed; contains genotype information) and ordinary PLINK (.ped, containing variant formation) files of 101 accessions of *P. latiovalifolius* and closely related *Phedimus* species for application in population genetics analyses.

### Phylogenetic and population genetic analyses

To determine the phylogenetic relationships between *P. latiovalifolius* and other *Phedimus* species in Korea, we conducted maximum likelihood (ML) analyses based on genome-wide 6642 SNPs in 101 samples using W-IQ-TREE^68,69^. Based on the Kim et al.’s nrDNA ITS tree^27^, *P. kamtschaticus* sampled from Sottongnyeong, Gangwon-do Province (PK_SDR), was used as the outgroup. Ultrafast bootstrap support (BS) was calculated from 1000 bootstrap replicates to determine clade robustness^70^. The best-fit substitution models were checked according to the Bayesian information criterion using ModelFinder^71^ implemented in IQ-TREE. Additionally, a species-partitioned SVDQuartets bootstrap consensus tree^72^ was generated using the default setting of 100,000 random quartets with the QFM quartet tree search algorithm and bootstrapping of 100 replicates in PAUP 4.0a169^72,73^. ML IQ-TREE and SVDQuartets phylogenetic trees were visualized using the online tool, the Interactive Tree of Life (iTOL) v6^74^.

The summary statistics of genetic diversity were presented by ‘populations’ in STACKS v1.48. Two populations of *P. latiovalifolius*, PL_DSS, and PL_DGR, were excluded due to a sample size of ≤ 3. In addition, the diversity statistics for three populations of *P. kamtschaticus*, PK_SDR, PK_CRS, and PK_KDB, were excluded because of their small population sizes (5, 3, and 4, respectively). Thus, 83 accessions were analyzed for genetic diversity, AMOVA, and *F*_st_. Analyses of molecular variance (AMOVA)^75^ and pairwise genetic differentiation between and among populations of *P. latiovalifolius* and congeneric *Phedimus* species were performed using GENODIVE ver. 3.5^76^. The pairwise correlation between geographic distance and genetic distance (Nei’s *G*_st_) among populations of *P. latiovalifolius* was calculated using Pearson’s correlation coefficient. The demographic history of *P. latiovalifolius* was inferred from folded SFSs using Stairway Plot 2^77^. The applied parameters were 6.075 × 10–9 per locus per year for mutation rate (*u*), 10 years for generation time (*g*), and 200 inferences that were used for the closely related genus, *Rhodiola* L. (Crassuraceae)^78^, as no data were found for *Phedimus*.

PLINK was used to estimate the population genetic structure and differentiation of the mountainous endemic *P. latiovalifolius* and congeneric *Phedimus* species. The eigenvalues and eigenvectors were calculated using principal component analysis (PCA) to examine the genetic similarities and relationships between individuals, and a PCA plot was drawn using the R statistical software (R 4.0.2). Population genetic structure was estimated by the maximum likelihood estimation of individual ancestries using ADMIXTURE ver. 1.3.0^38^. The best K value (the number of populations assumed for which the model had the best predictive accuracy) was chosen based on a low cross-validation error compared with other K values. Stacked bar plots of Q estimates of ancestry fractions were generated for target K values (best K and comparative K values) in R.

We used TreeMix v1.12^39^ to infer the admixture network among the populations of *P. latiovalifolius* and the congeneric *Phedimus* species. Based on the allele frequency of the SNP dataset, TreeMix first builds a Maximum Likelihood estimate of populations, including branch lengths (proportional to the amount of genetic drift between populations), and allows for migration between populations. Model fit was assessed by the degree to which migration edges (signifying gene flow between populations) reduced the residual genetic covariance among populations. To infer the best model, we tested models containing approximately 0 ~ 10 migration edges with ten replicates each. The dataset was filtered by removing the sites with missing data using VCFTOOLS v0.1.16^65^, and subsampled and bootstrapped with 80% of SNPs. The parameters of -global, -seed $RANDOM, and “-bootstrap -k 30” were used to run TreeMix to build ML trees by resampling blocks of 30 SNPs to ensure independency between blocks. Based on the output files produced by TreeMix, the OptM package^79^ in R was used to compare the model fit across models of different migration edges and replicates and to estimate the optimal number of migration edges. The R script “plotting_funcs.R” (https://github.com/joepickrell/pophistory-tutorial/blob/master/example2/plotting_funcs.R, accessed on February 16, 2023) was used to visualize the TreeMix graphs with migration results. The dataset was grouped by species and population to calculate all possible *f*_3_ (C, A, B) and *f*_4_ (A, B, C, D) statistics^80^ implemented in TreeMix to provide an analysis for comparison with the population admixture.

### Supplementary Information

Supplementary Information.

## Data Availability

The raw read sequences generated for this study can be found in the Sequence Read Archive (SRA) at the National Center for Biotechnology Information (NCBI) under the BioProject number PRJNA1037601 (Submission ID SUB13962522) with the title of “101 *Phedimus* GBS”.
