# Supplementary material for: Phylogenetic relationships and genetic diversity of the Korean endemic Phedimus latiovalifolius (Crassulaceae) and its close relatives
Source: Sci Rep. 2024 Jul 15;14:16255. doi: 10.1038/s41598-024-63272-9 (PMC11251145; doi:10.1038/s41598-024-63272-9)
Supplement: Supplementary file 1 — Supplementary Information. [file 41598_2024_63272_MOESM1_ESM.docx]

**Supplementary Materials**

**Phylogenetic relationships and genetic diversity of the Korean endemic *Phedimus latiovalifolius* (Crassulaceae) and its close relatives**

Myong-Suk Cho^1^, Yongsung Kim^2^, Seon-Hee Kim^3^, Ji-Hyeon Jeon^1^, JiYoung Yang^4^, and Seung-Chul Kim^1^*

**corresponding Seung-Chul Kim, sonchus96@skku.edu or* [*sonchus2009@gmail.com*](mailto:sonchus2009@gmail.com)

**1. Supplementary Figures**


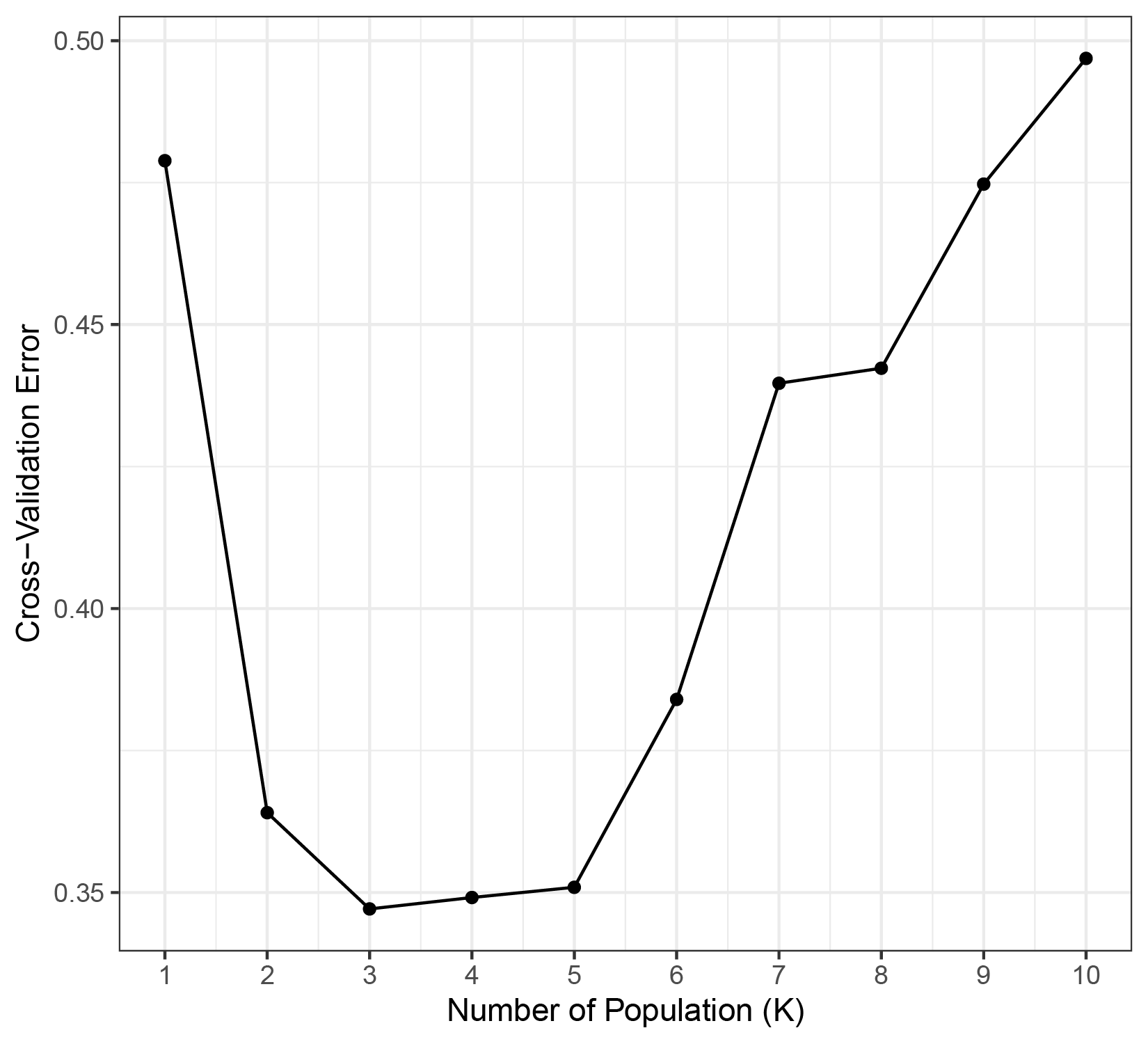


Supplementary Figure 1. Plot of Admixture cross validation error from K=1 through K=10. The best K value was chosen as K=3 with the lowest cross-validation error compared to other K values.


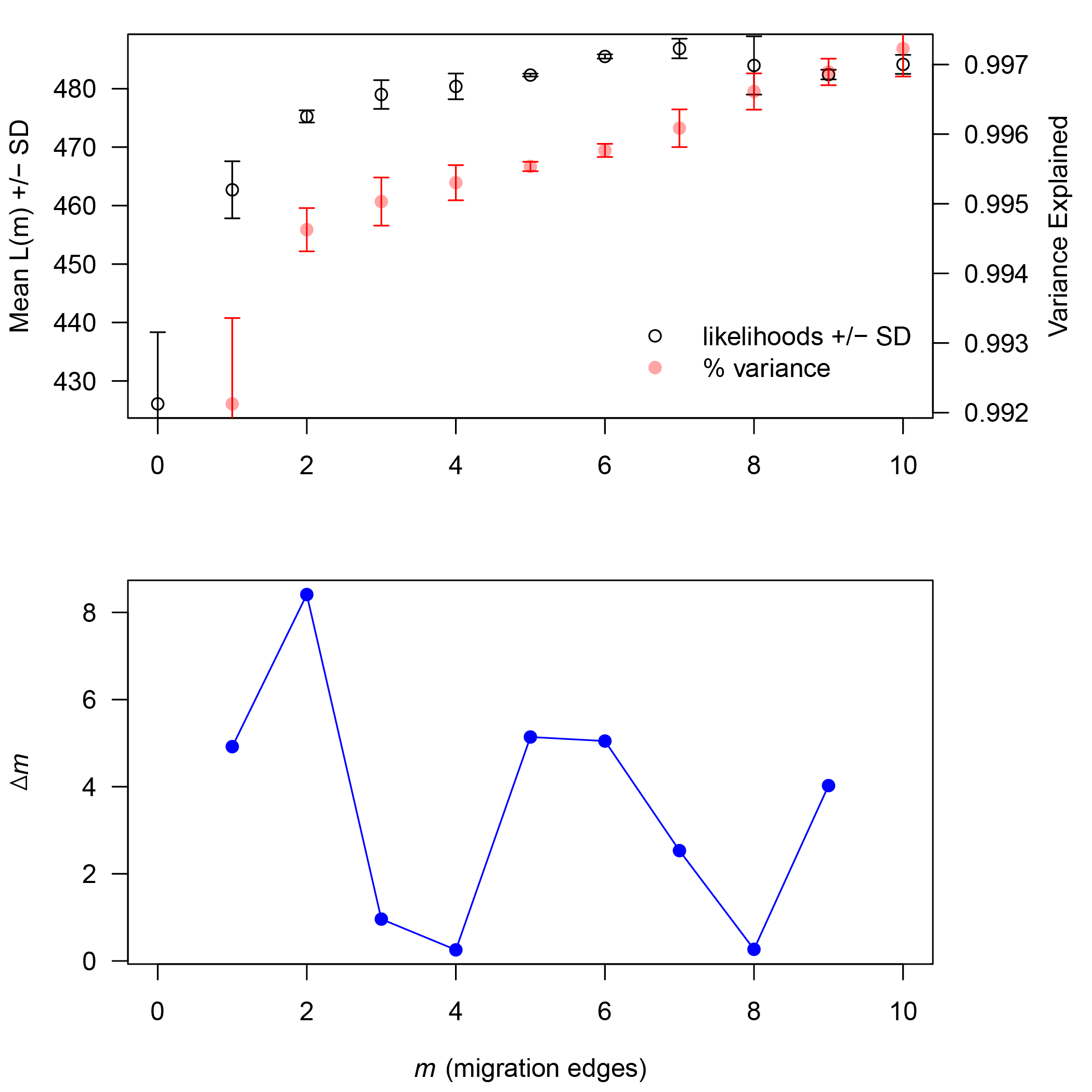
Supplementary Figure 2. The output produced by OptM to compare model fit across models of different migration edges. The optimal number of two migration edges (M2) was chosen with the highest value for Δ*m* (the second-order rate of change in likelihood across values of *m*) in optM from all migration events varying 0⁓10.


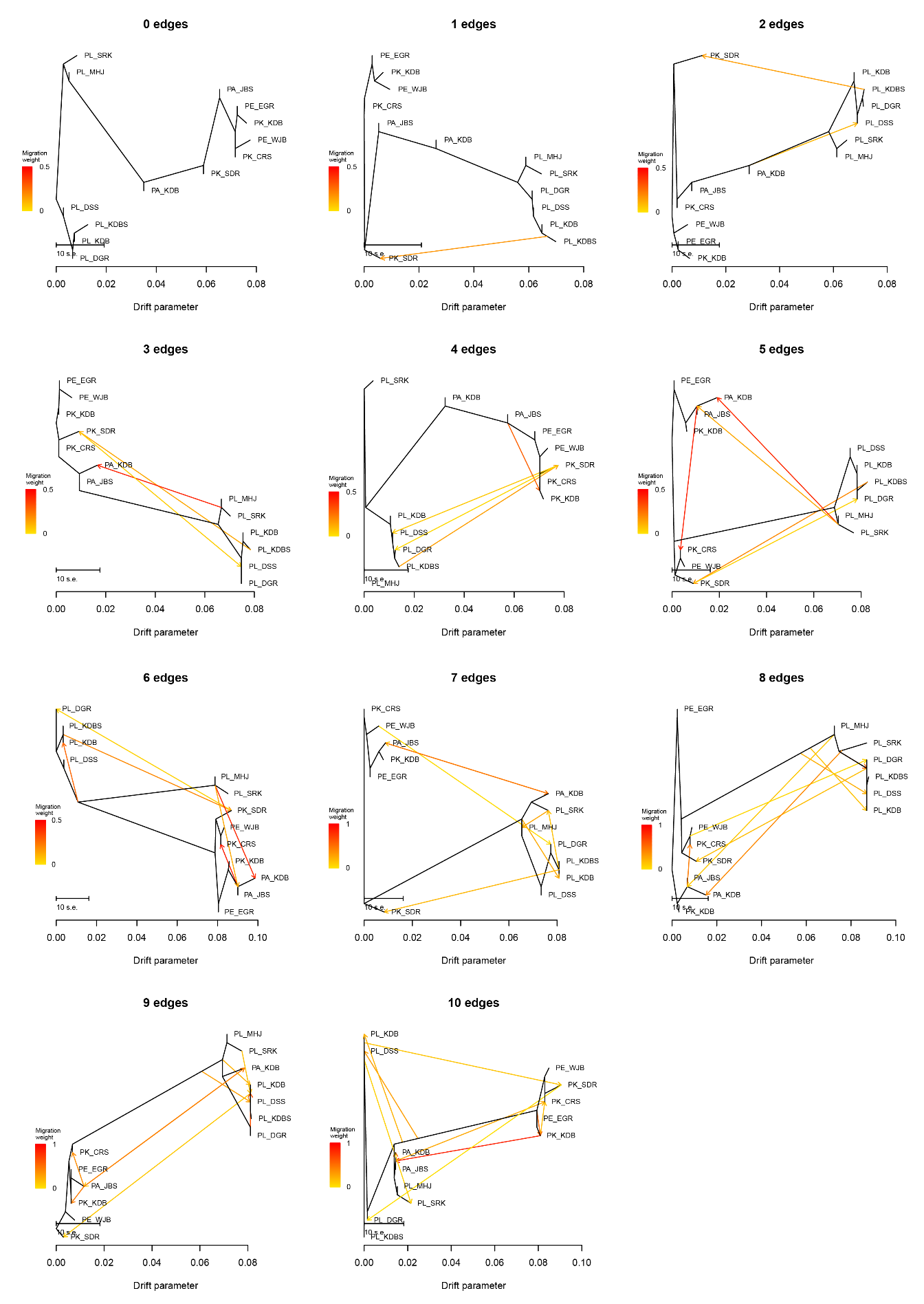


Supplementary Figure 3. Treemix iterations of various migration edges from M0 through M10.


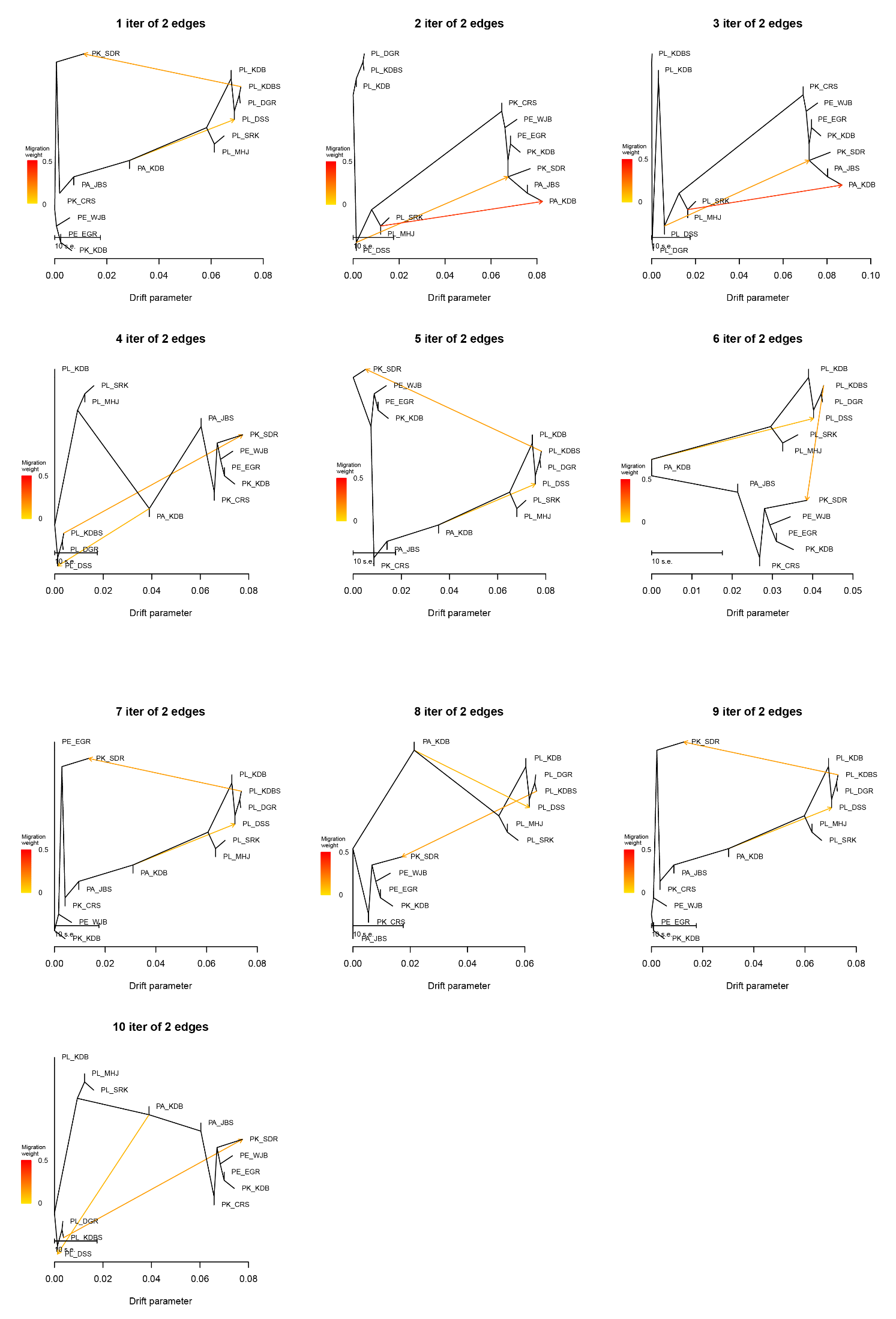


Supplementary Figure 4. Ten iterations of M2 with two migration events inferred by Treemix.

**2. Supplementary Tables**

Supplementary Table 1. Three-population test among the populations of *P. latiovalifolius* and congeneric *Phedimus* species.

| Population configuration | F3 | Standard error | Z-score |
| --- | --- | --- | --- |
| PL_MHJ; PL_SRK, PL_DGR | -0.00203 | 0.000435 | -4.67661 |
| PL_KDB; PL_DGR, PL_KDBS | -0.00157 | 0.000398 | -3.93866 |
| PL_MHJ; PL_SRK, PE_WJB | -0.00412 | 0.001137 | -3.62248 |
| PL_KDB; PL_KDBS, PK_SDR | -0.00263 | 0.00077 | -3.42316 |
| PL_KDB; PL_MHJ, PL_KDBS | -0.00278 | 0.00082 | -3.39081 |
| PL_MHJ; PL_SRK, PK_KDB | -0.00372 | 0.001111 | -3.35146 |
| Note: We performed f3-statistics (C; A, B) on all possible populations. Listed are at least one negative f3-score and Z-score less than -3; mixed populations C are those with significantly negative f3-statistics, not necessarily populations that were actually mixed historically. | | | |

Supplementary Table 2. Three-population test among the species of *P. latiovalifolius* and congeneric *Phedimus* species.

| Population configuration | F3 | Standard error | Z-score |
| --- | --- | --- | --- |
| PK; PL, PE | -0.00489 | 0.000653 | -7.49663 |
| PA; PL, PE | -0.00575 | 0.001081 | -5.31596 |
| PK; PA, PE | -0.00226 | 0.000715 | -3.15626 |
| PA; PK, PL | -0.00311 | 0.001442 | -2.15795 |
| Note: We performed f3-statistics (C; A, B) on four *Phedimus* species; PL (*P. latiovalifolius*), PA (*P. aizoon*), PE (*P. ellacombeanus*), and PK (*P. kamtschaticus*). Listed are at least one negative f3-score and Z-score less than -3; mixed species C are those with significantly negative f3-statistics, not necessarily populations that were actually mixed historically. | | | |

Supplementary Table 3. Four-population test among *P. latiovalifolius* and congeneric *Phedimus* species.

| Population configuration | F3 | Standard error | Z-score |
| --- | --- | --- | --- |
| PK, PA; PL, PE | -0.01572 | 0.00299 | -5.25558 |
| PK, PE; PA, PL | -0.00264 | 0.000816 | -3.23182 |
| PK, PL; PA, PE | -0.01835 | 0.002601 | -7.05595 |
| Note: We performed f4-statistics (A, B; C, D) on four *Phedimus* species; PL (*P. latiovalifolius*), PA (*P. aizoon*), PE (*P. ellacombeanus*), and PK (*P. kamtschaticus*). | | | |
